# Predicting Outcomes and Optimizing Therapy in Thrombotic Thrombocytopenic Purpura: Insights on Caplacizumab Use from a Romanian Hematology Center

**DOI:** 10.3390/jcm14228211

**Published:** 2025-11-19

**Authors:** Diana Oana Preda, Mihai Emanuel Himcinschi, Adelina Vlad, Florentina Adriana Gauianu, Daniel-Nicolae Murariu, Oana-Ruxandra Croitoru, Daniel Coriu, Sorina Nicoleta Badelita

**Affiliations:** 1 Hematology and Bone Marrow Transplant Center, Fundeni Clinical Institute, 022328 Bucharest, Romaniadaniel_coriu@yahoo.com (D.C.);; 2 Department of Biochemistry, Victor Babes University of Medicine and Pharmacy, 300041 Timisoara, Romania; 3 Department of Hematology, Carol Davila University of Medicine and Pharmacy, 022328 Bucharest, Romania

**Keywords:** thrombotic thrombocytopenic purpura prediction factors, Adamts13, fluorescence resonance energy transfer

## Abstract

**Background:** Thrombotic thrombocytopenic purpura (TTP) is a rare and life-threatening thrombotic microangiopathy requiring prompt diagnosis and treatment. This retrospective single-center study analyzed 31 adult patients diagnosed between 2013 and 2024 (PLASMIC score ≥ 5, ADAMTS13 activity < 10%), aiming to characterize their clinical profiles and assess the impact of caplacizumab. **Methods:** Baseline laboratory parameters (platelet count, LDH, creatinine, hemoglobin, number of plasmapheresis sessions, number of hospitalization days, number of days in intensive care, and days required to recover platelet count) were included in statistical analysis to predict diverse outcomes, such as respiratory distress, infection, major neurological manifestations, gastrointestinal involvement, or refractoriness/exacerbation. Sixteen patients underwent treatment with caplacizumab in addition to plasmapheresis (PEX) and corticosteroids, while the remainder received PEX and corticosteroids alone. **Results:** Our predictive models proved noteworthy, providing results with ROC values ranging from 0.80 to 0.90 (*p* < 0.01). Caplacizumab was associated with faster platelet recovery (4 days vs. 7 days), fewer PEX sessions, shorter hospital stays, and a significantly lower incidence of refractoriness or exacerbation (*p* < 0.05). Inter-group analysis confirmed a significant reduction of overall resource use (*p* < 0.05). **Conclusions:** Early caplacizumab use improved outcomes and optimized resource utilization. This real-world study suggests that routinely available laboratory markers at presentation can help predict outcomes and guide early clinical decisions in centers without rapid ADAMTS13 testing.

## 1. Introduction

Thrombotic thrombocytopenic purpura (TTP) is a subtype of thrombotic microangiopathies (TMAs), a group of disorders characterized by obstruction of the microvasculature due to the formation of intraluminal thrombi. Clinically, TMAs typically present with microangiopathic hemolytic anemia and thrombocytopenia, collectively known as MAHAT [1,2]. TTP is a rare and life-threatening disorder with an annual incidence ranging from 1.5 to 6 cases per million adults and a prevalence of approximately 10 cases per million individuals [1,3,4]. The condition primarily affects adults, with about 90% acute episodes occurring during this stage of life [5,6].

Most cases of TTP are acquired and result from an autoimmune response that leads to a severe deficiency in the ADAMTS13 enzyme, which is responsible for cleaving von Willebrand factor multimers. Inadequate ADAMTS13 activity determines the accumulation of ultra-large von Willebrand factor multimers, followed by platelet aggregation and microvascular thrombosis [1,2,7,8]. A rare inherited form of the disease known as congenital TTP (cTTP) or Upshaw–Schulman syndrome is caused by mutations in the ADAMTS13 gene [9,10], and typically presents in childhood, often before the age of 10 [1,3,11,12].

TTP is characterized by a severe reduction in platelet count, microangiopathic hemolytic anemia (low hemoglobin, elevated bilirubin, elevated LDH), neurological manifestations such as confusion or seizures, renal involvement, and, in some cases, fever or gastrointestinal symptoms [2,10] Although these manifestations are largely nonspecific, they may precede acute complications, including respiratory distress, infections, and treatment refractoriness [1,13,14].In the absence of prompt testing of ADAMTS13 activity, rapid recognition of these signs becomes critical. International studies [1-3,13] have already validated scores such as PLASMIC or the French score for the triage of patients with severe enzymatic deficiency.

The present study makes an important contribution by providing a predictive model based on accessible biological parameters such as platelet count, creatinine, LDH, and hemoglobin. This model enables real-time therapy guidance and risk prediction, including the decision to initiate early caplacizumab therapy.

Prompt initiation of treatment for thrombotic thrombocytopenic purpura is essential. Current strategies in the acute phase primarily involve plasma exchange and corticosteroids, which have decreased mortality rates from approximately 90% to under 20% [3,15,16,17]. More robust studies are required, especially to evaluate newer therapies like caplacizumab and cost-effective options in resource-limited settings [18,19,20]. Therapeutic plasma exchange (TPE) should be initiated as soon as TTP is confirmed or strongly suspected and continued until complete remission is achieved (typically defined by normalization of cell counts and full resolution of clinical symptoms) [17,21,22,23].

Between 30% and 60% of individuals diagnosed with TTP experience relapse, with a mortality rate of 5–20%. Historically, adjunctive treatments, including immunosuppressive agents (e.g., vincristine, azathioprine, and cyclosporine A), high-dose intravenous immunoglobulin, and splenectomy have been used in relapsed or refractory cases [24,25,26]. In the early 2000s, rituximab, a monoclonal chimeric antibody targeting CD20 on mature B cells, was introduced for patients with recurrent or refractory TTP [24,27,28]. Additionally, several observational cohort studies and a recent meta-analysis suggest that rituximab administration during the acute phase reduces both relapse rates and mortality compared to a control group [26,29,30].

Bortezomib, a proteasome inhibitor, has also been explored for its ability to directly target plasma cells and lower antibody levels. However, evidence regarding its effectiveness remains limited [26,31].

Unlike rituximab, whose clinical effects may take days to weeks, caplacizumab acts quickly by targeting the A1 domain of von Willebrand factor, preventing platelet adhesion [26]. Caplacizumab, a humanized single-variable-domain immunoglobulin, has demonstrated effectiveness and safety in the phase 2 TITAN and phase 3 HERCULES trials [32,33]. A detailed analysis of these studies indicates that caplacizumab significantly reduces the risk of mortality and refractoriness. Further research also supports its benefit in early administration during both initial and recurrent episodes [33,34,35]. Although associated with a risk of hemorrhagic events, these are typically mild and do not require medical intervention [33,36].

This unicentric retrospective study, conducted in a Romanian reference hematology unit, aimed to provide insights into the therapeutic efficacy of caplacizumab and to refine predictive approaches tailored to our patient population. Specifically, we sought to identify laboratory parameters predictive of clinical outcomes and to examine the relationships between therapeutic strategies, baseline clinical presentation, and laboratory profiles. We hypothesized that routine laboratory data could guide diagnosis and optimize disease management.

## 2. Materials and Methods

### 2.1. Ethical Approval

The study was conducted at the Hematology and Bone Marrow Transplant Center (HBMTC) of the Fundeni Clinical Institute, Bucharest, in accordance with the Declaration of Helsinki. All data were anonymized before analysis. The study protocol was approved by the Ethics Committee of the Fundeni Clinical Institute, Bucharest, Romania (20397/20.05.2025). Informed consent for participation in clinical research was obtained from all patients upon admission.

### 2.2. Subjects and Study Design

The study was designed as a retrospective, single-center analysis conducted at the Fundeni Hematology and Bone Marrow Transplantation Center in Bucharest. It included consecutive patients diagnosed with TTP between 2013 and 2024 (*n* = 31). A definitive diagnosis required a PLASMIC score ≥ 5 and ADAMTS13 activity < 10% (initially assessed by a semi-quantitative method, and later by a fully automated FRET method).

Participants were divided into two groups according to current clinical practice: PEX + corticosteroids and PEX + caplacizumab. At diagnosis and during follow-up, demographic, clinical, and biological variables were collected, including sex, blood pressure, complete blood count (platelet count, hemoglobin, leukocytes, etc.), schistocyte evaluation, creatinine, LDH, Coombs test (performed only at diagnosis for differential diagnosis), ALT/AST, total and direct bilirubin, and ADAMTS13 activity. Additionally, data on the number of PEX sessions, length of hospitalization, days spent in intensive care, and time to platelet normalization were recorded.

Outcome definitions were established as follows: clinical response, exacerbation, and relapse. Refractoriness was defined as a platelet count <50 × 10^9^/L and LDH > 1.5 × ULN despite 5 PEX sessions. Time to platelet normalization was defined as the interval between the first PEX session and the first day with a platelet count ≥150 × 10^9^/L.

Only five patients had a PLASMIC score below 6. The overall occurrence of infection, shortness of breath, neurological manifestations, gastrointestinal involvement, and refractoriness is detailed in Table 1.

The PLASMIC score was used to evaluate the likelihood of severe ADAMTS13 deficiency in patients with suspected TTP. This scoring system assigns one point for each of the following seven criteria: platelet count < 30 × 10^9^/L, evidence of hemolysis (reticulocytosis, elevated indirect bilirubin, undetectable haptoglobin, negative Coombs test), absence of active cancer within the last year, no history of solid organ of stem cell transplantation, mean corpuscular volume (MCV) < 90 fL, international normalized ratio (INR) < 1.5, and serum creatinine < 2 mg/dL. The total score is interpreted as follows: 0–4 points, low risk (<5%); 5–6 points, intermediate risk (50–60%); 6–7 points, high risk (>90%) [3,11,17,37].

### 2.3. ADAMTS13 Activity Assays; Transition to Fully Automated FRET-Based Testing

ADAMTS13 activity was measured using fluorescence resonance energy transfer (FRET), a sensitive method based on the non-radiative transfer of energy between two fluorophores in nanometer-scale proximity. The transfer efficiency is inversely proportional to the sixth power of the distance between them, allowing rapid and accurate quantification of enzymatic activity. Compared to older semi-quantitative methods, FRET offers improved reliability and significantly reduces processing times.

In 2022, our laboratory transitioned from the semi-quantitative assay, which provided broad activity intervals sufficient for treatment decisions but lacked precision for monitoring therapy or early relapse, to a fully automated FRET-based system. This new system measures absolute ADAMTS13 activity values ranging from 0 to 160% with a resolution of 0.001% and delivers results within 27 min, reducing turnaround time from four hours to one hour—an important advantage in emergency settings.

The transition involved equipment installation and calibration, staff training, and comparative testing against the previous method. We validated the new assay on a cohort of 20 patients and assessed inter-patient variability following therapy. Despite higher costs, the automated assay offers superior sensitivity at low ADAMTS13 levels, which is critical for detecting relapses during the high-risk 30-day period post-remission, particularly when activity falls between 10% and 15%. It is important to note that elevated plasma bilirubin, high lipid levels, severe hemolysis, and high doses of unfractionated heparin can interfere with the assay and should be considered during result interpretation [11].

### 2.4. Therapeutic Plasma Exchange

All patients underwent therapeutic plasma exchange (TPE), initiated within the first 4 h of clinical suspicion of TTP, following emergency management protocols [3,5]. Central venous access was established using a jugular or femoral catheter capable of withstanding flow rates exceeding 60 mL/min. TPE was performed using continuous centrifugation-based apheresis platforms or membrane-based systems with selective permeability. The necessary calculation for determining the total plasmatic volume was performed using the following formula: plasma volume (L) = 0.07 × body weight (kg) × (1 − hematocrit), as described by George et al. [9,13]. 

### 2.5. Statistical Analysis

All the data collected from the hospital’s register were generated and processed using GraphPad Prism for Windows (v.9) software (GraphPad Software, San Diego, CA, USA).

Statistical tests were employed to examine the relationships between biological test results and clinical manifestations, as well as the impact of different therapies on the clinical course of patients with TTP. A multiple logistic regression model was used to assess whether routine laboratory parameters, such as platelet count, bilirubin, creatinine, LDH, and hemoglobin, could predict the recurrence of acute complications, including respiratory distress, infections, gastrointestinal or neurological involvement, and the risk of refractoriness or exacerbation. Model performance was validated using receiver operating characteristic (ROC) curves, with predictive efficiency considered acceptable at an area under the curve (AUC) >0.7 and *p*-value < 0.01. The same multiple logistic regression was used in order to determine if therapeutic outcomes, such as number of PEX sessions, number of hospitalization days, number of days in intensive care, and days required to recover platelet count could serve as indicators for the prediction of post-treatment refractoriness/exacerbation.

To show how disperse data was for different indicators, a variation coefficient (VC) was calculated.

To compare the impact of the two therapeutic protocols (with and without caplacizumab), a one-way ordinary ANOVA analysis was performed, followed by a multicomparison post-test. This analysis was used to compare group means for variables including the number of plasma exchange (PEX) sessions, length of hospitalization, days spent in intensive care, and time to platelet recovery. Fisher’s exact test, supplemented by Monte Carlo approximation due to the small number of events, was used to evaluate the association between treatment protocols and the risk of refractoriness [3,18,20]

The Mann–Whitney test was used to evaluate whether there were any differences between the two main study groups.

A *p*-value below 0.05 was considered statistically significant for all tests and is indicated by “*” in tables and figures.

## 3. Results

### 3.1. Patient Characteristics

Our initial cohort comprised 31 patients, of whom 16 were treated with a combination of plasma exchange (PEX) and caplacizumab, while the remainder received PEX and corticosteroid therapy alone. The group displayed a predominant female incidence of 64% and a median age of 43 years, aligning with known demographic trends for autoimmune aTTP [3,13] The means and standard deviations, representation—mean *±* standard deviation, of continuous variables in the two working groups are presented in Table 1 and Table 2.

By evaluating the distribution of patients treated with caplacizumab versus PEX, the only significant difference observed was in the number of hospitalization days (*p* = 0.004), with a mean of 10.4 vs. 20.8 days (Table 2).

### 3.2. Subgroup Analysis Based on Treatment Type

The two treatment-based subgroups were analyzed for the coefficient of variation in the following clinical variables: number of PEX sessions (VC = 56.5% for the caplacizumab group and VC = 56.6% for the PEX group), hospitalization days (VC = 43.1% for the caplacizumab group and VC = 63.3% for the PEX group), days in intensive care (VC = 265% for the caplacizumab group and VC = 214% for the PEX group), and days required to recover the platelet count (VC = 60.7% for the caplacizumab group and VC = 51.8% for the PEX group). To further evaluate the differential effects of the two treatment protocols on these variables, a variation analysis was performed in the PEX group compared to the caplacizumab group, revealing a statistically significant difference between treatments for the number of days spent in hospital (*p* < 0.05).

**Figure 1:**
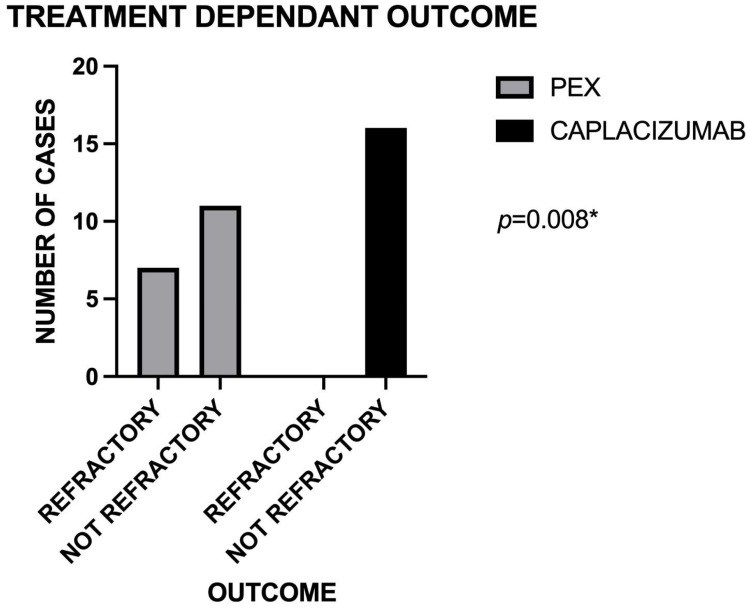
Treatment-dependent outcomes for refractoriness (* = statistical significant).

We assessed whether the treatment type was associated with predisposition to refractoriness, and the contingency analysis yielded a *p*-value of 0.008 (Figure 1). Our results suggest that adding caplacizumab to plasma exchange and corticosteroids may reduce refractoriness and improve clinical outcomes compared to plasma exchange and corticosteroids alone (0% refractory in the caplacizumab group vs. 20.59% in the PEX group). However, no significant correlation was found between clinical manifestations and disease severity based on the individual PLASMIC score parameters (*p* < 0.05).

### 3.3. Predictive Modeling of Clinical Outcomes

**Figure 2:**
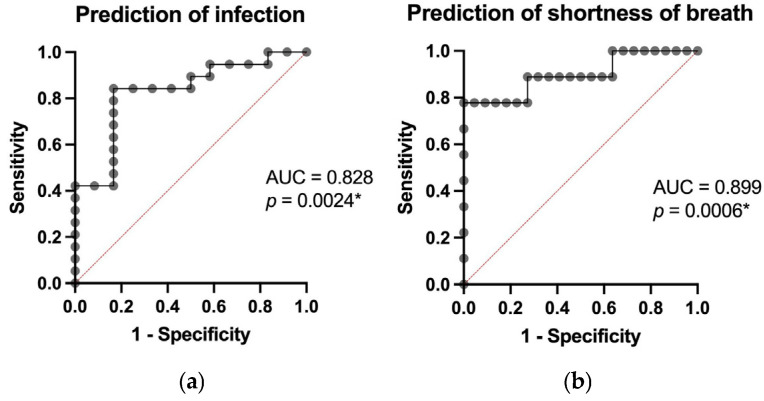
Prediction of infection (**a**) and shortness of breath (**b**), using continuous values of age, hemoglobin, leucocyte count, platelet count, creatinine, total/direct/indirect bilirubin, and LDH (* = statistical significant).

**Figure 3:**
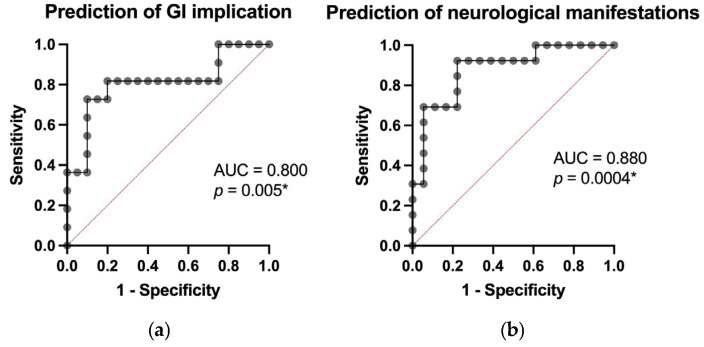
Prediction of GI implication (**a**) and neurological manifestation (**b**), using continuous values of age, hemoglobin, leucocyte count, platelet count, creatinine, total/direct/indirect bilirubin, and LDH (* = statistical significant).

Multiple predictive models (based on data from all 31 patients) were used to evaluate whether clinical outcomes, including infection, shortness of breath, gastrointestinal involvement, major neurological symptoms, treatment refractoriness, exacerbation, and sex differences, could be predicted using continuous variables obtained through laboratory tests. The model’s performance, expressed as the area under the ROC curve (AUC) with corresponding *p*-values, was as follows: infection, AUC = 0.828 (*p* = 0.0024), shortness of breath, AUC = 0.899 (*p* = 0.0006), gastrointestinal involvement, AUC = 0.800 (*p* = 0.005), major central neurological symptoms, AUC = 0.880 (*p* = 0.0004) using continuous values of age, hemoglobin, leucocyte count, platelet count, creatinine, total/direct/indirect bilirubin, LDH, taken at the moment of diagnosis and refractoriness or exacerbation, AUC = 0.870 (*p* = 0.0005). These results are illustrated in Figure 2 and Figure 3.

These results point towards a strong capacity to anticipate acute complications, with AUC values ranging from 0.800 to 0.899, indicating high discriminative power. They also highlight the complex biological profile of thrombotic thrombocytopenic purpura (TTP), in which low platelet counts, endothelial dysfunction, oxidative stress [15], and increased tissue permeability contribute to the development of multi-organ complications.

### 3.4. Prediction of Treatment Response and Post-Treatment Outcomes

**Figure 4:**
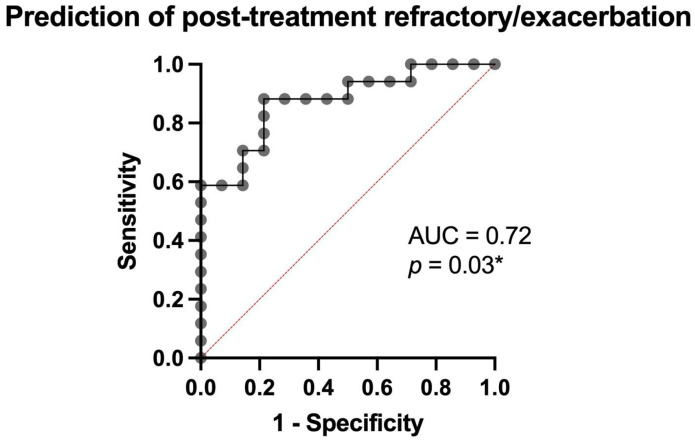
Prediction of post-treatment refractoriness/exacerbation using the number of PEX sessions, number of hospitalization days, number of days in intensive care, and days required to recover platelet count (* = statistical significant).

We applied the same predictive models for post-treatment outcomes (exacerbation or disease relapse), considering treatment with only PEX and corticosteroid therapy as indicative of an unfavorable prognosis. Predictor variables included the number of PEX sessions, number of hospitalization days, number of days in intensive care, and days required to recover platelet count. The model achieved an area under the ROC curve of 0.72 (*p* = 0.03) (Figure 4). These findings highlight the complex and multifaceted biological profile of thrombotic thrombocytopenic purpura (TTP), in which low platelet counts, endothelial dysfunction, oxidative stress [11] and increased tissue permeability contribute to the development of multi-organ complications.

## 4. Discussion

Our cohort, while limited in size, illustrates the classical clinical profile of acquired thrombotic thrombocytopenic purpura. Post-treatment outcomes, like therapeutic refractoriness, defined as failure to respond to first-line treatment, were brought into light by our predictive model that incorporates routinely available laboratory parameters. Variables such as age, hemoglobin, leucocyte count, platelet count at diagnosis, creatinine, bilirubin, and LDH levels proved sensitive in indicating a suboptimal response. These findings support early therapeutic escalation, particularly the early administration of caplacizumab in patients presenting with a platelet count below 20,000/microliter, high LDH or troponin levels, renal failure, severe neurological presentation at diagnosis, to reduce associated complications and patient mortality [3,9,11,38]. Another aspect that we focused on is providing prognostic factors based on the type of therapy and procedural variables, such as the number of PEX sessions. Our analysis suggests that the ongoing evolution is highly influenced by dynamic factors such as clearance of inhibitors, the response to glucocorticoids, and the immunological status that cannot be fully resumed at admission. This underlines the need for longitudinal assessment and flexible therapeutic strategies.

If the goal in clinical practice is early disease detection and rapid identification of patients at risk for severe complications, our algorithm, despite being developed from a limited cohort, may serve as a valuable decision support tool, particularly for centers lacking access to ADAMTS13 activity testing. This is especially relevant in intensive care units where time-sensitive decisions are critical. Such approaches have the potential to help identify patients at higher risk of complications and refractoriness, thereby guiding the timely administration of anti-CD20 drugs and other targeted therapies [3,11,17].

By dividing our cohort into two groups—patients treated with PEX alone and those receiving PEX plus caplacizumab—we observed a reduction in intragroup variability among the caplacizumab group for some of our data. The analysis shows that the variability of clinical indicators differs between the two treatment groups. The number of PEX sessions was similarly heterogeneous in both groups (CV 56.5% vs. 56.6%). In contrast, the duration of hospitalization was more homogeneous in patients treated with caplacizumab (CV 43.1%) than in those treated with PEX alone (CV 63.3%), suggesting more predictable outcomes in the first group. Intensive care days showed very high variability in both groups (CV 265% and 214%), due to many zero values and isolated cases with prolonged hospitalization. Time to platelet recovery had moderate-high variability in both groups (60.7% in caplacizumab and 51.8% in PEX), slightly higher in the caplacizumab group, likely reflecting individual differences in response.

Moreover, inter-group variation analysis confirmed a statistically significant impact of treatment type on clinical outcomes (*p* < 0.05) for days spent in hospital). Caplacizumab was associated with accelerated hematologic remission and reduced the global need for PEX utilization, consistent with data reported by the Hercules and Titan trials [32,33] These results validate the role of caplacizumab in reducing hospitalization duration and thus, procedural burden. Within the PEX-only group, a single patient stands out due to prolonged hospitalization because of an initial misdiagnosis of ITP. The low Plasmic score and atypical presentation delayed the TTP diagnosis, which was confirmed only after Adamts13 testing, highlighting the crucial need for rapid, accurate diagnostics [3,11,17]. 

From a pathophysiological perspective, the clinical profile of the patient group—characterized by severe thrombocytopenia, hemolytic anemia, and marked increases in LDH, reflects heightened microthrombotic activity, in which vWF multimers promote platelet aggregation by binding to glycoprotein Ib, thereby accelerating intravascular hemolysis and platelet consumption. In this context, caplacizumab rapidly interrupts microvascular thrombus formation, which is clinically manifested as faster platelet normalization and a reduced duration of hospitalization, without necessarily increasing the number of PEX sessions. The correlation between primary biological markers and differences in patient progress between therapeutic groups supports a likely causal effect of antagonizing the vWF–GPIb interaction on the rapid resolution of thrombotic microangiopathy. This mechanism explains why the risk of refractoriness or exacerbation is lower when anti-vWF therapy is initiated promptly, in parallel with PEX and corticosteroid treatment. Similar results were also presented in the Capla 1000+ project [39].

Our statistical analysis has put into perspective a significant association between first-line treatment choice and refractoriness risk (*p* < 0.05). This supports the early inclusion of caplacizumab in therapeutic protocols. Diving into the mechanistic of caplacizumab, it neutralizes ultra-large vWF multimers, preventing platelet aggregation persistence and limiting inflammatory amplification, processes that are central to TTP pathophysiology.

Even though caplacizumab requires higher upfront costs, the reduction in hospital stay and PEX sessions may finally compensate for these expenses—an aspect with particular relevance in institutions with constrained resources. Our data plead for an early use of caplacizumab in all centers with available funding. In resource-limited centers or in cases where the drug is contraindicated, combination therapy with PEX and corticosteroids remains essential.

This study shares similarities with the HERCULES trial in terms of baseline characteristics, including age and gender distribution, as well as LDH and platelet levels. In our cohort, the time to platelet recovery was four days in the caplacizumab group versus seven days in the PEX group, slightly less rapid than HERCULES, which reported a three-day median. The number of PEX sessions was comparable (five in both groups), reinforcing the external validity of our findings despite the absence of randomization or placebo control [32,33]. Unlike the randomized and double-blind HERCULES study, our single-center, retrospective analysis reflects real-world TTP management in a Romanian reference center. The lack of immediate access to ADAMTS13 testing prompted us to develop pragmatic solutions for triaging patients and guiding therapy based on routine laboratory and clinical markers. This aspect, not directly addressed by the HERCULES trial, is particularly relevant for centers without access to high-performance molecular diagnostic infrastructure.

Moreover, the present investigation uniquely utilized statistical analyses to assess risk for complications and refractoriness, offering an individualized, resource-adapted approach to care. Additionally, it was extensively used to determine the predictive values of common biological markers (LDH, platelet count, hemoglobin, and creatinine) in identifying acute aggravations such as respiratory distress, infections, or major neurological manifestations, as well as the risk of post-treatment refractoriness or exacerbations. Our study contributes significantly to personalizing initial therapeutic decisions based on the early biological profile, particularly in the absence of rapid ADAMTS13 activity results. Another original contribution is the transition from a semi-quantitative to a fully automated, quantitative ADAMTS13 testing method. This shift significantly reduced the time needed to obtain results and increased sensitivity in identifying patients at risk of relapse after remission, illustrating how regional technological advancements can directly influence clinical decisions and patient prognosis. A major component of the study lies in its monocentric nature, characterized by a consistent basic approach (PEX + corticosteroids), uniform definitions, and identical endpoints/biomarkers, which ensure valid comparisons across different time periods. The division into two groups (PEX vs. PEX + caplacizumab) and the observed decline in intragroup variability after the introduction of caplacizumab, together with a proportionate number of PEX sessions, indicate not merely a timing artifact but a genuine therapeutic effect. In addition, the proposed algorithm is based on readily available routine indicators, remaining applicable even where access to ADAMTS13 testing is delayed. Overall, the progressive developments over the years do not represent a major bias but rather contextualize the scientific advances and strengthen the practical applicability of the results. Nevertheless, as a pilot study, secondary external validation remains recommended.

The results of the predictive models suggest that a set of readily available biomarkers, including platelets, hemoglobin, LDH, bilirubin, and creatinine, can adequately differentiate patients at risk for acute events, providing a valuable tool for early risk stratification even before ADAMTS13 results are available. For practical implementation, we propose the following approach: early initiation of caplacizumab, PEX, and corticosteroids in patients with a high predicted risk, followed by reassessment at 48–72 h to monitor the dynamics of these biomarkers. Increasing or decreasing trends serve as dynamic indicators of microthrombosis control. Subsequent integration of ADAMTS13 levels and, when applicable, inhibitor titers can further refine patient management decisions. Importantly, in addition to discrimination, model calibration and time-dependent sensitivity analyses enhance the robustness of predictions and mitigate potential confounding induced by differential timing of treatment initiation.

This pilot study is limited by its retrospective, single-center design and relatively small sample size, with data originally collected for clinical rather than research purposes, introducing potential selection bias. The absence of randomization and placebo control increases the risk of residual confounding and limits the ability to draw robust causal inferences. Additionally, the relatively small sample size reduces statistical power and increases the likelihood of false negatives. Applying complex statistical analyses to a small dataset may also yield unstable parameter estimates. From the same point of view, we could mention that the application of statistical analyses to a rather small sample group could also yield unstable parameter estimates. Furthermore, the transition from a semi-quantitative to quantitative Adamts13 testing during the study period could affect the precision of phenotype classification and introduce variability in time-to-diagnosis-related outcomes. However, such transitions are common with evolving diagnostic infrastructure and represent a necessary adaptation to technological progress [11,17,39].

Despite these limitations, the observed efficacy signals for caplacizumab, including reduced PEX use and faster remission, offer exploratory evidence that should be confirmed in larger or prospective studies. Until such validation is available, the proposed recommendations and predictive algorithm should be considered as clinical guidance rather than definitive standards.

## 5. Conclusions

Our findings point towards the feasibility of predicting acute complications and therapeutic refractoriness in a TTP using routinely available laboratory parameters such as hemoglobin, platelet count, and LDH. The introduction of caplacizumab significantly reduced PEX sessions, hospitalization time, ICU stays, and platelet recovery time, improving both therapeutic predictability and overall patient outcomes.

While routine laboratory testing was moderately predictive of post-treatment relapse, more reliable prognostic value lies in serial ADAMTS13 activity and inhibitor testing, as well as treatment-specific response variables.

Additional research is needed to validate our findings in larger, multicenter, prospective studies. Future directions include the integration of machine learning algorithms and cost-effectiveness analysis to refine treatment strategies and enhance personalized TTP management, particularly in resource-limited settings.

## Figures and Tables

**Table 1:** Means, standard deviations, and correlation between caplacizumab and PEX groups (Mann–Whitney) of patient characteristics and baseline laboratory parameters.

	Age (yr)	HEMOGLOBIN (g/dL)	LEUKOCYTES (×10^3^/µL)	PLATELETS (×10^3^/µL)	CREATININE (μmol/L)	BILIRUBIN (μmol/L)	LDH (U/L)	PLASMIC SCORE (Points)
OVERALL (*n* = 31)	44 *±* 11.16	7.79 *±* 1.33	11.3 *±* 4.48	16.5 *±* 11.87	1.2 *±* 0.56	2.8 *±* 1.8	1925 *±* 1142	6 *±* 0.74
CAPLACIZUMAB GROUP (*n* = 16)	44 *±* 11.25	7.9 *±* 1.388	10.5 *±* 3.85	14.8 *±* 11.6	1.1 *±* 0.5	2.3 *±* 1.1	2000 *±* 1280	5.9 *±* 0.68
PEX GROUP (*n* = 15)	44.7 *±* 11.46	7.6 *±* 1.28	12.2 *±* 5	18 *±* 12.1	1.2 *±* 0.57	3.4 *±* 2.3	1837 *±* 1011	6 *±* 0.7
Mann–Whitney (*p*)	0.89	0.6	0.47	0.34	0.9	0.22	0.98	0.13

**Table 2:** Means and standard deviations of patient outcomes .

	PEX SESIONS (No.)	HOSPITAL STAY (Days)	ICU STAY (Days)	PLATELET RECOVERY (Days)
OVERALL (*n* = 31)	6.1 *±* 3.46	15 *±* 10.94	1.3 *±* 3.2	6 *±* 3.73
CAPLACIZUMAB (*n* = 16)	5.6 *±* 3.1	10.4 *±* 4.5	1.3 *±* 3.4	4.6 *±* 2.8
PEX GROUP (*n* = 15)	6.6 *±* 3.7	20.8 *±* 13.22	1.4 *±* 3.1	7.7 *±* 4
Mann–Whitney (*p*)	0.48	0.004 *	0.66	0.23

* = statistical significant.

## Data Availability

All data can be made available by contacting our corresponding author.

## Abbreviations

The following abbreviations are used in this manuscript:

FRET	Fluorescence resonance energy transfer
iTTP	Immune thrombotic thrombocytopenic purpura
TPE	therapeutic plasma exchange
CPP	Cryoprecipitate-poor plasma
TTP	Thrombotic thrombocytopenic purpura
TMAs	Thrombotic microangiopathies
vWF	von Willebrand factor
FFP	Fresh frozen plasma
